# Cytoreductive nephrectomy in the era of immune‐checkpoint inhibitors: back to the future?

**DOI:** 10.1111/bju.70168

**Published:** 2026-02-11

**Authors:** Rocco Simone Flammia, Riccardo Campi, Eugenio Bologna, Riccardo Bertolo, Costantino Leonardo, Fabio Calabrò, Daniele Amparore, Giuseppe Simone, the Young Academic Urologists (YAU) Renal Cancer Working Group

**Affiliations:** ^1^ Uro‐Oncology Program IRCCS Regina Elena National Cancer Institute Rome Italy; ^2^ Advanced Surgical Technologies PhD programme Sapienza University of Rome Rome Italy; ^3^ Urological Robotic Surgery and Renal Transplantation Unit, Department of Experimental and Clinical Medicine Careggi Hospital University of Florence Florence Italy; ^4^ European Association of Urology (EAU) Young Academic Urologists (YAU) Renal Cancer Working Group Arnhem The Netherlands; ^5^ Urology Unit, Department of Surgery, Dentistry, Pediatrics and Gynecology University of Verona – Azienda Ospedaliera Universitaria Integrata Verona (AOUI) Verona Italy; ^6^ Division of Urology, Department of Surgery Candiolo Cancer Institute, FPO‐IRCCS Candiolo Italy; ^7^ Department of Oncology University of Turin Orbassano Italy

**Keywords:** cytoreductive nephrectomy, immune‐checkpoint, immunotherapy, renal neoplasm, tyrosine kinase inhibitor

## Abstract

**Objective:**

To investigate the role of cytoreductive nephrectomy (CN) in metastatic renal cell carcinoma (mRCC) treated with immune‐checkpoint inhibitors (ICIs).

**Methods:**

A narrative review was carried out using PubMed and searching for English articles published from January 2015 to May 2025.

**Results:**

After the screening process, 12 retrospective studies comparing outcomes in patients with mRCC treated with ICI‐based regimens, with or without CN (either upfront or deferred) were deemed eligible. Of those, six indicated a survival benefit for patients undergoing CN in combination with ICIs with hazards ratios ranging from 0.19 to 0.63, a finding that remains consistent within the upfront CN subgroup. However, the included studies’ retrospective nature, inherent selection, and immortal time biases limit definitive conclusions. Ongoing phase III randomised trials, NORDIC‐SUN (ClinicalTrials.gov identifier: NCT03977571) and Southwest Oncology Group (SWOG)‐1931 (also known as PROBE; NCT04510597), are evaluating the role of deferred CN after initial ICI therapy, while the role of upfront CN in the ICI era will be likely elucidated by SEVURO‐CN (NCT05753839) trial.

**Conclusion:**

Our findings highlight reconsidering the importance of CN in the ICI era, potentially driven by the influence of tumour burden on anti‐cancer immunity and the limited efficacy of ICIs against primary tumours. Future research, ideally through randomised trials involving patients suitable for safe surgery, should aim to clarify the optimal timing of CN in the context of ICI therapy.

**Abbreviations**
(d)(u)CN: (deferred) (upfront) cytoreductive nephrectomy
FDA: United States Food and Drug Administration
HR: hazard ratio
ICI: immune‐checkpoint inhibitor
IL‐6: interleukin 6
IPTW: inverse propensity of treatment weighting
mRCC: metastatic RCC
NCDB: National Cancer Database
OS: overall survival
PFS: progression‐free survival
RCT: randomised controlled trial
RN: radical nephrectomy
TKI: tyrosine kinase inhibitor

## Introduction

Immune‐checkpoint inhibitor (ICI)‐based regimens have emerged as the cornerstone of treatment for metastatic RCC (mRCC), demonstrating significant and durable disease regression in a substantial proportion of patients [1]. Notably, as seen in studies from the targeted therapy era, the majority of patients enrolled in ICI clinical trials—over 80%—had undergone radical nephrectomy (RN) years before initiating systemic treatment. This preponderance of patients having undergone RN, coupled with the absence of randomisation based on nephrectomy status, limits the ability of these studies to accurately isolate the impact of cytoreductive nephrectomy (CN) on overall survival (OS) in the ICI era [2]. To address this gap, we conducted a literature review focusing on studies published since 2015, when the United States Food and Drug Administration (FDA) approved the first ICI regimen for mRCC [3].

## Methods

We conducted a literature search of the PubMed electronic databases for the purpose of writing a narrative review based on the following PICO:

- Patient: mRCC
- Intervention: CN
- Comparator: only ICI‐based therapy
- Outcomes: OS

The following search string was implemented: (‘cytoreductive nephrectomy’[Title/Abstract] OR ‘cytoreductive nephrectomy’[Title/Abstract] OR ‘Nephrectomy’[MeSH]) AND (‘immune checkpoint inhibitor*’[Title/Abstract] OR ‘PD‐1 inhibitor*’[Title/Abstract] OR ‘PD‐L1 inhibitor*’[Title/Abstract] OR ‘CTLA‐4 inhibitor*’[Title/Abstract] OR ‘nivolumab’[Title/Abstract] OR ‘pembrolizumab’[Title/Abstract] OR ‘atezolizumab’[Title/Abstract] OR ‘ipilimumab’[Title/Abstract] OR ‘Immune Checkpoint Inhibitors’[MeSH]) AND (‘renal cell carcinoma’[Title/Abstract] OR ‘kidney cancer’[Title/Abstract] OR ‘renal cancer’[Title/Abstract] OR ‘RCC’[Title/Abstract] OR ‘Carcinoma, Renal Cell’[MeSH]). All English written article published between Janu 2015 to May 2025 were included.

## Evidence Synthesis

We identified 12 retrospective studies comparing outcomes between patients undergoing CN—either upfront (uCN) or deferred (dCN)—plus ICI‐based regimens and those treated with ICI‐based regimens alone [4, 5, 6, 7, 8, 9, 10, 11, 12, 13, 14, 15] (Table 1). Among the studies examined, 10 were classified as ‘pure’ retrospective studies [4, 5, 6, 7, 8, 11, 12, 13, 14, 15], three of which were based on national or international registries, while only two were *post hoc* analysis of randomised controlled trials (RCTs) despite survival estimates from Grimm et al. [10] being included in the pooled analysis from Fallah et al. [9], which collected individual‐patient data from five RCTs, namely JAVELIN Renal 101 (ClinicalTrials.gov identifier: NCT02684006), KEYNOTE‐426 (NCT02853331), CheckMate 9ER (NCT03141177), CLEAR (NCT02811861), and IMmotion151 (NCT02420821). [Correction added on 25 March 2026, after first online publication: ‘19’ has been corrected to ‘10’ in the preceding sentence.]

**Table 1:** General information and characteristics of the included studies.

References	Type of study	Years	Inclusion criteria	*N*	Comparison*	Statistical strategy	Median FU, months	Type of ICI regimen considered
Singla et al., 2020 [4]	Retrospective (NCDB‐based)	2015–2016	*De novo* mRCC Only ICI‐based therapy ± uCN or dCN	391	**Overall cohort** **CN + ICI (*n* = 221) vs ICI alone (*n* = 170)**	MV	14.7	ICI‐ICI or ICI monotherapy
					Subgroup uCN (*n* = 197) vs dCN (*n* = 24)		14.7	
Yoshino et al., 2022 [14]	Retrospective (multicentre)	2016–2021	*De novo* mRCC First‐line Nivo/Ipi	41	Overall cohort ICI + dCN (*n* = 7) vs uCN + ICI (*n* = 21) vs ICI alone (*n* = 13)	None	12.0	ICI‐ICI (Nivo/Ipi)
Ghatalia et al., 2022 [5]	Retrospective (US Flatiron Health EHR National Database)	2011–2020	*De novo* mRCC with predominant clear cell histology ST alone; uCN or dCN + ST; CN alone	1910	Overall cohort uCN (*n* = 605) vs systemic alone (*n* = 972)	MV IPTW Landmark analysis Time‐varying regression		ICI‐ICI; ICI alone; ICI‐TKI
					Subgroups ‐ uCN (*n* = 605) vs dCN (*n* = 142) **‐ uCN + ICI (*n* = 116) vs ICI alone (*n* = 285)**			
Hahn et al., 2023 [12]	Retrospective (multicentre: MD Anderson, MSKCC)	2013–2021	*De novo* mRCC with sarcomatoid and/or rhabdoid dedifferentiation Received ICI‐based regimen as first or second line	157	**Overall cohort** **ICI + CN (*n* = 118) vs ICI alone (*n* = 39)**	MV Time‐dependent Cox regression DAG	33.9	ICI‐ICI; ICI‐TKI; ICI mono
					Sensitivity analysis uCN + ICI (*n* = 89) vs ICI alone (*n* = 39)			
Porta et al., 2023 [15]	Retrospective (ARON‐1 real‐world study)	2016–2022	*De novo* mRCC First‐line immune combinations	651	Overall cohort ICI + CN (*n* = 255) vs ICI alone (*n* = 396)	Landmark analysis	31.5	ICI‐ICI; ICI‐TKI
Hara et al., 2023 [11]	Retrospective (multicentre)	2018–2021	*De novo* mRCC Treated with Nivo/Ipi	54	**Overall cohort** **uCN + ICI (*n* = 21) vs ICI alone (*n* = 33)** *dCN (*n* = 3) excluded	Propensity score matching (1:1) MV	15.7	ICI‐ICI (Nivo/Ipi)
Gross et al., 2023 [6]	Retrospective multicentre analysis (bi‐centre)	2000–2020	*De novo* or metachronous mRCC + ICI‐based therapy without RN prior to mRCC diagnosis	367	Overall cohort CN + ICI (*n* = 232; 202 uCN and 30 dCN) vs ICI alone (*n* = 135)	MV	28.4	ICI‐ICI or ICI alone
					**Subgroup analysis** **CN + first‐line ICI (*n* = 47) vs first‐line ICI alone (*n* = 56)**		12.5 ICI alone 19.2 in ICI + CN	
Bakouny et al., 2023 [7]	Retrospective multicentre analysis	2009–2020	*De novo* or metachronous mRCC + ICI‐based therapy or TT without nephrectomy prior to mRCC	4639	Sub‐cohorts: **‐ uCN + ICI (*n* = 234) vs ICI alone (*n* = 203)** ‐ uCN + TT (*n* = 2326) vs TT alone (*n* = 1876)	MV IPTW MCS Interaction analysis for prior CN	24 TT group 12 ICI group	ICI‐ICI, ICI‐other agents, or ICI alone
Grimm et al., 2023 [10]	*Post hoc* analysis of the Javelin Trial	2016–2017	*De novo* mRCC	412	Sub‐cohorts: **‐ uCN + ICI (*n* = 126) vs ICI alone (*n* = 72)** ‐ uCN + TT (*n* = 147) vs TT alone (*n* = 67)	MV Interaction analysis for prior CN	Minimum FU duration of 28	ICI‐TKI
Fallah et al., 2024 [9]	*Post hoc* pooled analysis of Javelin, Keynote‐426, CheckMate 9ER, CLEAR, IMmotion151	2015–2019	*De novo* mRCC	981	Overall cohort **uCN + ICI (*n* = 596) vs ICI alone (*n* = 385)**	MV	20	ICI‐TKI
Park et al., 2024 [13]	Retrospective (CKCis Database)	2014–2023	*De novo* mRCC First‐line IO‐based ST (IO/IO or IO/TKI)	588	Overall cohort ICI alone (*n* = 331) vs uCN + ICI (*n* = 215) vs ICI + dCN (*n* = 42)	MV	17–30 (varies by group)	ICI‐ICI; ICI‐TKI
Yildirim et al., 2025 [8]	Retrospective national cancer database	2018–2020	*De novo* mRCC receiving uCN + ST or ST alone	872	Overall cohort uCN + ICI/TKI (*n* = 130) vs ICI/TKI (± dCN) (*n* = 742)	MV IPTW		ICI‐ICI, ICI‐TKI, or ICI alone
					**Subgroup analysis** **uCN + ICI (*n* = 63) vs ICI (± dCN) (*n* = 370)** uCN + TKI (*n* = 67) vs TKI (± dCN) (*n* = 372)			

DAG, directed acyclic graph; IO, immunotherapy; Ipi, ipilimumab; MCS, multivariate confounding score; MV, multivariable analysis; Nivo, nivolumab; ST, systemic therapy; TT, targeted therapy.

**Fig. 1:**
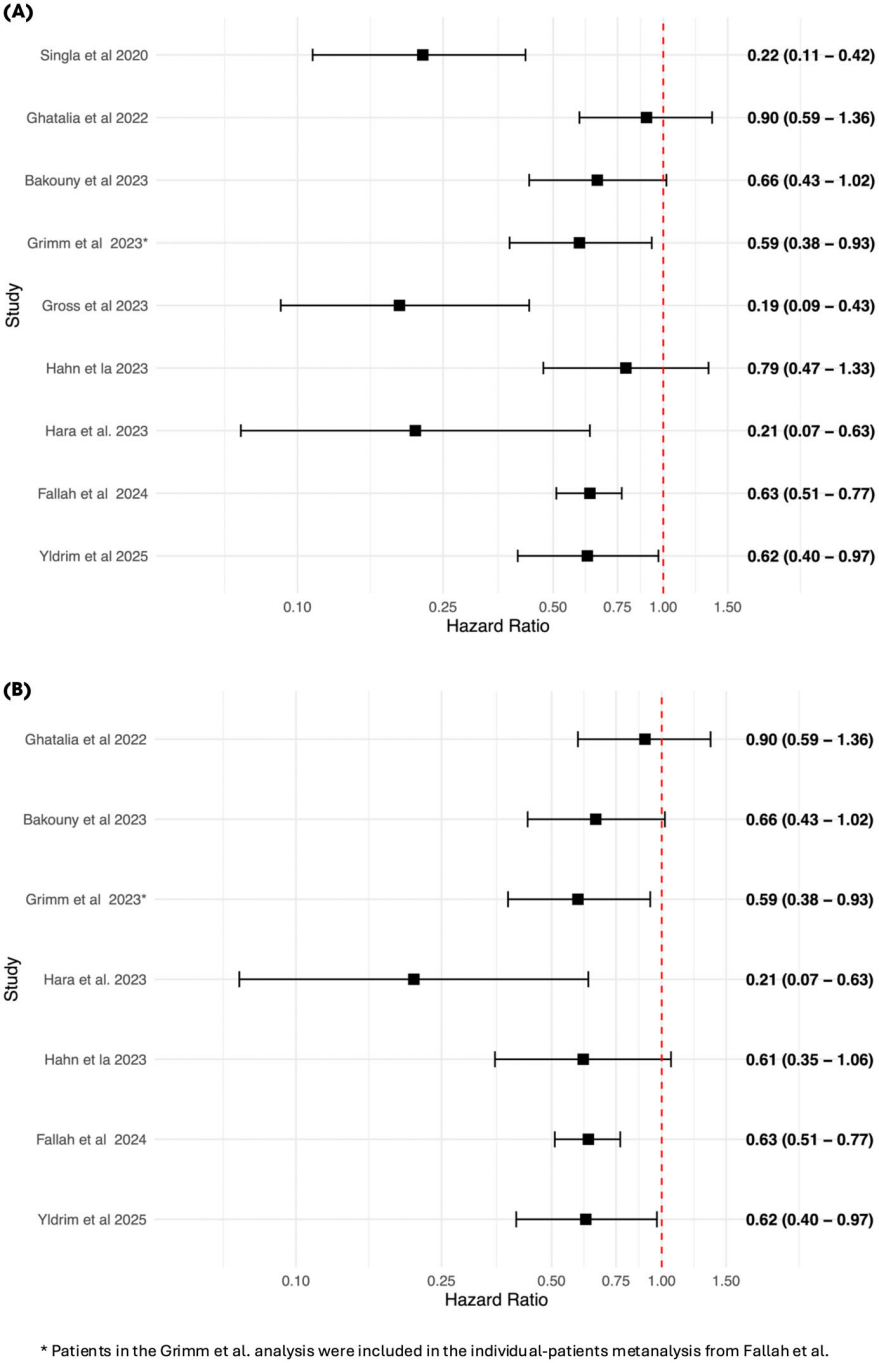
Forest plots depicting the pooled HR for OS comparing CN + ICI vs ICI alone in the overall cohort **(A)**, and in the subgroup of uCN + ICI vs ICI alone **(B)**. A HR <1 favours CN + ICI vs ICI alone. *Patients in the Grimm et al. [10] analysis were included in the individual‐patients meta‐analysis from Fallah et al. [9]. [Correction added on 25 March 2026, after first online publication: Figure 1 has been updated for accuracy.]

* Bolded cohorts are those for whom Cox regression HRs of interest were provided and depicted in the Fig. 1.

Initially, Singla et al. [4] analysed 391 patients with mRCC from the National Cancer Database (NCDB; 2015–2016) reporting an improved OS for uCN followed by ICI relative to ICI alone (hazard ratio [HR] 0.22, 95% CI 0.12; 0.42). Subsequently, Gross et al. [6] by analysing a bi‐centric North America cohort spanning 2000–2020 recorded a protective role for CN (HR 0.19, *P* < 0.001, Fig. 1) in the ICI‐based subgroup (*n* = 102) in terms of OS with most patients treated with uCN (44 vs three dCN). Park et al. [13], analysing data from the Canadian Kidney Cancer Information System (CKCis) on 588 patients treated with first‐line ICI, reported superior OS for patients undergoing either uCN (HR 0.68, 95% CI 0.47–0.97, *P* = 0.03) or dCN (HR 0.30, 95% CI 0.13–0.68, *P* = 0.004). Similarly, the ARON‐1 study [15] (NCT05287464) provided evidence on OS benefit of CN in a multicentre cohort of >600 patients with mRCC from different geographical areas (median OS: not reached vs 24 months, *P* < 0.001). Additionally, the FDA‐approved *post hoc* analysis of Fallah et al. [9] reported a protecting effect on OS (adjusted HR 0.63, 95% CI 0.51–0.77) and progression‐free survival (PFS; adjusted HR 0.71, 95% CI 0.59–0.85) of uCN vs ICI alone. These results echoed previous findings from Hara et al. [11] reporting OS and PFS benefit in mRCC treated with uCN (median PFS 3.4 vs 0.8 months, *P* = 0.016; median OS 38.4 vs 12.6 months, *P* = 0.002) after propensity score‐matching. More recently, Yildirim et al. [8] published a study showing a protective effect of CN in mRCC after inverse probability of treatment weighting (IPTW) adjustments (HR 0.62, 95% CI 0.40–0.97). In contrast with these results, Ghatalia et al. [5] did not find a statistically significant association between uCN and OS in mRCC ICI‐based treated patients from the Flatiron Health database (*n* = 433). However, this finding might be affected using regression with time‐varying covariates (HR 0.90, 95% CI 0.59–1.36). Indeed, they observed a wide difference in IPTW adjusted median OS between uCN group vs those receiving ICI‐based therapy alone (40.2 vs 15.2 months, respectively). Another study with conflicting results was published by the International Metastatic RCC Database Consortium. Here, uCN was associated with significantly OS benefit in the ICI subgroup (*n* = 437) in comparison with ICI alone after multivariable adjustment (HR 0.61, 95% CI 0.41–0.90, *P* = 0.013). However, a loss of statistical significance after IPTW adjustments (HR 0.66, 95% CI 0.43–1.02) was recorded. Notably, both the study from Bakouny et al. [7] and Grimm et al. [10] failed to show any statistically significant interaction between uCN and ICI‐based vs tyrosine kinase inhibitor (TKI) regimens questioning whether cohort composition in terms of patients and tumour characteristics rather that the drug used may affect results. Last but not least, caution is warranted when considering CN for aggressive histological subtypes. Hahn et al. [12] conducted a critical analysis of patients with sarcomatoid and/or rhabdoid mRCC treated with ICIs. Notably, when using time‐dependent covariates to adjust for immortal time bias, failed to record statistically significant survival benefit associated with CN (HR 0.79; *P* = 0.37) in this high‐risk population.

Another important finding emerging from these studies regards objective and complete response rates in favour of uCN vs ICI‐based therapy alone (Fallah et al. [9]: 60% vs 46% and 13% vs 1%; Gross et al. [6]: 46.9% vs 26.8% and 19.2% vs 0%; Hara et al. [11]: 47.6% vs 27.3% and 14.3% vs 0%, Park et al. [13]: 41.4 vs 32% and 7 vs 0%). Despite these differences may somehow be influenced by selection bias, it deserves attention the high rate of objective and complete response among uCN patients. Indeed, Bickley et al. [16] observed the prognostic relevance of an exceptional response to ICIs, defined radiographically as complete or near‐complete (>80%) response at metastatic sites among patients with mRCC.

Taken together, the currently available scientific evidence is insufficient to definitively accept or reject the role of CN + ICI in mRCC.

This uncertainty stems from several key limitations and challenges in interpreting the available data. In addition to the well‐known biases inherent to retrospective studies, two specific biases significantly limit the interpretation and depth of discussion in this area of interest: the selection bias for surgical patients and the so‐called ‘immortal time’ bias affecting patients deemed ‘candidates’ for surgery. Although the selected studies report the use of statistical techniques aimed at mitigating these biases—such as multivariable models, IPTW, interaction studies, time‐varying covariate analysis, and the utilisation of multivariable confounding scores—the authors themselves acknowledge that, despite their reliability, these methods carry an inherent risk of residual confounding. This residual confounding can influence the analyses and, consequently, the associated hypotheses. In addition, among the studies analysed, a notable heterogeneity exists regarding the initial inclusion criteria for patients. While 10 studies assessed the effect of surgery exclusively in the ‘*de novo*’ mRCC setting, the other two included both ‘*de novo*’ and ‘metachronous’ patients. This variability introduces additional complexity, as these studies do not account for the potential impact of prior treatments for the primary tumour—administered before the development of metastases—on both the efficacy of systemic therapy and the criteria for surgical candidacy.

Despite these limitations, these studies have shed new light on the potential role of CN in the ICI era, contrasting with the negligible influence previously attributed to CN in the targeted‐therapy era, based on CARMENA (NCT00930033) [17] and SURTIME (NCT01099423) findings [18]. The advent of ICI‐based therapy in the mRCC setting necessitates a critical re‐evaluation of CN based on various theories and observations. One key rationale is that a high tumour burden negatively impacts anticancer immunity, potentially mediated by interleukin 6 (IL‐6) released by cancer cells, which modulates tumour‐associated macrophages and myeloid‐derived suppressor cells [19]. By decreasing IL‐6 levels, CN may enhance the efficacy of ICI‐based therapy in mRCC. Similarly, Goswami et al. [20] demonstrated that CN decreases KDM6B‐expressing immune‐suppressive myeloid cells in the peripheral blood. Additionally, the low likelihood of achieving a complete response in the primary tumour with ICIs could lead to the accumulation of resistant clones, which may promote further dissemination and resistance to ICI‐based regimen [21, 22]. Moreover, while evidence suggests no significant difference in the efficacy of ICI‐based therapy compared to sunitinib regardless of nephrectomy status, it is noteworthy that most registration trials for ICI therapies predominantly included patients who had already undergone removal of their primary tumour [23].

To date, two phase III and a phase IV randomised trials are investigating the role of CN in this specific patient population. The NORDIC‐SUN trial (NCT03977571) aims to compare clinical outcomes between patients receiving or not receiving dCN following a combination of nivolumab and ipilimumab in patients with mRCC classified as intermediate‐ or poor‐risk according to the International mRCC Database Consortium (IMDC) criteria [24]. Similarly, the Southwest Oncology Group (SWOG)‐1931 (also known as PROBE; NCT04510597) trial is evaluating the impact of dCN in patients with mRCC treated with one of the FDA‐approved ICI‐based first‐line combinations [25]. In the PROBE trial, patients will be randomised to undergo dCN 10–14 weeks after initiating ICI‐based regimens if they achieve stable disease, partial response, or if the investigator determines they are deriving clinical benefit from systemic therapy. Conversely, the ITALIC‐RCC (NCT06903312) phase IV randomised trial will compare clinical outcomes of dCN vs radiation therapy vs no local treatment in patients with mRCC following initial clinical benefit from ICIs‐based combinations. Notably, none of these studies is currently investigating the role of uCN in mRCC in the ICIs era. What exactly drove this trend is unclear, but this choice is likely attributable to the results of the CARMENA and SURTIME trials, as well as the therapeutic efficacy of the approved ICI‐based regimens. First, in the sunitinib‐only arm of the CARMENA trial, a total of 40 patients (18%) eventually underwent dCN [26]. These patients demonstrated a longer median OS compared to those who did not undergo surgery (48.5 vs 15.7 months). Second, the SURTIME trial found no significant difference in the PFS between patients treated with sunitinib prior to CN and those who underwent uCN followed by sunitinib [27]. Third, new ICI‐based regimens have reported a notable proportion (10%) of complete responses at metastatic sites, while showing limited efficacy on primary tumours, likely due to higher tumour heterogeneity [22]. Fourth, Khandwala et al. [28] by analysing found that major pathological response, defined as percentage of residual viable tumour <10% at primary tumour site among mRCC treated with ICI, was significantly associated with improved PFS (HR 0.05, 95% CI 0.01–0.41, *P* = 0.005) and OS (HR 0.07, 95% CI 0.01–0.88, *P* = 0.039), thus suggesting a role of dCN to guide treatment discontinuation in selected patients. Taken together, this highlights the potential benefits of dCN, which allows for the immediate initiation of systemic therapy while reserving surgery for patients who respond to initial treatment, thereby avoiding the loss of a critical therapeutic window.

Nonetheless, the studies discussed in this review seem to suggest that uCN still remains a common and effective treatment for well‐selected patients (Fig. 1B). In this regard, Makrakis et al. [29] by meta‐analysing individual‐patient data reported survival benefit for uCN (HR 0.52, 95% CI 0.40–0.69) relative to ICI alone. Notably, a recent systematic review highlighted a persistent interest in the role of surgical timing for CN, despite lack of high‐quality evidence [30]. In this regard, Singla et al. [4] reported no difference in terms of OS (HR 0.25; 95% CI 0.03–1.83), 30‐day readmission (0% vs 4.6%, *P* = 0.602), and positive surgical margin (0% vs 14.7%, *P* = 0.050) for dCN vs uCN in the ICI regimes. Yoshino et al. [14] failed to show survival differences and recorded comparable estimated blood loss and transfusion rates between the two arms (42% vs 75%, *P* = 0.12), despite longer operative time in uCN (250 vs 192 min, *P* = 0.03). Similarly, Park et al. [13] not only did not report statistically significant OS difference between dCN vs uCN in their three‐arm comparison but also report similar complications rates between the two approaches (14% vs 21%, *P* > 0.5). Additionally, a recent population‐based analysis in the United States by Ditonno et al. [31] observed that patients undergoing uCN experience lower rates of overall, medical, and surgical 30‐day postoperative complications compared to their dCN counterparts. This underscores the importance of performing CN at the optimal time to minimise surgical morbidity while preserving oncological benefit. In this regard, biomarker, such as circulating tumour DNA [32] or next generation imaging such as girentuximab [33] may play a role in selecting patients for CN and targeting optimal timing.

Finally, the southern‐Korean SEVURO‐CN phase II/III randomized trial (NCT05753839) represents the only attempt to investigate the role and timing of CN, by comparing uCN vs dCN vs ICI‐based therapy alone among patients with intermediate poor‐risk synchronous mRCC eligible for surgery [34]. Additionally, the CYTOSHRINK (NCT04090710), a phase II RCT, will evaluate the effect of stereotactic body radiation therapy to the primary kidney tumour as an alternative to uCN in patients who are ineligible for surgery.

## Conclusion

While ongoing trials are expected to provide valuable insights into the role of dCN in the ICI era, we acknowledge that identifying the subset of patients who might derive the greatest benefit from uCN remains an unresolved dilemma. Ideally, other studies similar to the SEVURO‐CN phase II/III randomised trial, addressing this question should enrol only patients for whom safe surgery, preferably performed using minimally invasive techniques when feasible, can be achieved, minimising complications that might delay the initiation of systemic therapy.

## Disclosure of Interests

The authors declare there are no conflicts to disclose.
